# Inferring General Relations between Network Characteristics from Specific Network Ensembles

**DOI:** 10.1371/journal.pone.0037911

**Published:** 2012-06-06

**Authors:** Stefano Cardanobile, Volker Pernice, Moritz Deger, Stefan Rotter

**Affiliations:** 1 Bernstein Center Freiburg, University of Freiburg, Freiburg im Breisgau, Germany; 2 Faculty of Biology, University of Freiburg, Freiburg im Breisgau, Germany; University of Namur, Belgium

## Abstract

Different network models have been suggested for the topology underlying complex interactions in natural systems. These models are aimed at replicating specific statistical features encountered in real-world networks. However, it is rarely considered to which degree the results obtained for one particular network class can be extrapolated to real-world networks. We address this issue by comparing different classical and more recently developed network models with respect to their ability to generate networks with large structural variability. In particular, we consider the statistical constraints which the respective construction scheme imposes on the generated networks. After having identified the most variable networks, we address the issue of which constraints are common to all network classes and are thus suitable candidates for being generic statistical laws of complex networks. In fact, we find that generic, not model-related dependencies between different network characteristics do exist. This makes it possible to infer global features from local ones using regression models trained on networks with high generalization power. Our results confirm and extend previous findings regarding the synchronization properties of neural networks. Our method seems especially relevant for large networks, which are difficult to map completely, like the neural networks in the brain. The structure of such large networks cannot be fully sampled with the present technology. Our approach provides a method to estimate global properties of under-sampled networks in good approximation. Finally, we demonstrate on three different data sets (*C. elegans* neuronal network, *R. prowazekii* metabolic network, and a network of synonyms extracted from Roget’s Thesaurus) that real-world networks have statistical relations compatible with those obtained using regression models.

## Introduction

The development of models for the topology underlying complex interactions in natural systems has attracted much attention in recent research [1]–[3]. The structure of such systems exerts strong influence on their dynamics [4]–[6]. To characterize the network structure quantitatively, usually statistical measures are introduced. Indeed, in many cases parametric families of network models exist that can replicate specific statistics observed in real networks and also explain how these statistics arise. Classical examples are the emergence of a giant connected component in percolation phenomena [7], and the power-law degree distributions observed in real-world networks [8]. Dynamical systems on networks have recently received much attention. The influence of certain structural features on dynamical properties, like synchronizability [9], [10] and controllability [11], [12] has been analyzed with the help of particular network models. This fact calls for an evaluation of the efficiency of existing network models in sampling the space of real-world networks. In fact, it is unlikely that a small number of standard models can reproduce the variability of networks observed in nature, but this problem is rarely addressed in the literature. To circumvent this problem we base our analysis on several different network models to avoid singular relations that hold only for specific cases. Remaining relations among different structural network features can then with much greater certainty be assumed to hold generally. In particular, we take advantage of two recently developed advanced network models, multifractal networks [13], [14] and equilibrium random networks [15]. These new classes encompass networks of greater structural diversity in the statistical ensemble than for example Erdös-Rényi graphs or small-world networks and might therefore be more suitable to assess the influence different network properties have on each other.

For instance, let us assume that a dependence between two features can be expressed in terms of an explicit mathematical relation. An example is the correspondence between node degrees and Laplacian eigenvalues [16]. Such a relation would, of course, manifest itself in a correlation between these features across different network realizations for any network ensemble. However, such analytical results are hard to find in general. It is easier to identify suitable candidates from correlations that are present for certain network ensembles. In this way also less general relations, which hold only for a subset of networks, can be detected. Such relations are useful if they apply to certain classes of empirical networks. In order to search for non-trivial relations that are also general, an ensemble of networks is needed that does not introduce relations which hold only in this specific ensemble. For example the study of ring networks together with random networks suggests that high clustering coefficients are associated with large characteristic path lengths. The analysis of small world networks [9], however, demonstrates that this relation is not valid in general.

At the same time, the ensemble should produce very variable networks, in the sense that the values of the interesting features should have a broad distribution, such that relations can be observed with high significance. As an empirical quantity to judge the quality of network ensembles with respect to these two demands, we introduce an entropy measure based on the distribution of features.

As a first main result, we conclude that multifractal networks and equilibrium random networks are the most variable ones with respect to the generated feature entropy. They present a good sampling basis, as only weak correlations between different graph properties are imposed by their construction principle.

The issue of whether global, in particular spectral, properties of networks are predictable from local statistical properties has been debated in the scientific community with both negative [17] and positive [16] results. Our second main result is that global network properties, also of spectral nature, are indeed statistically linked to network properties on a local level, and that these relations are also relevant for real-world networks. This is achieved using multivariate linear regression on an appropriate set of regressors among the local features.

In particular, we study three different networks: a synonym network extracted from Roget’s Thesaurus [18], the metabolic network of the bacterium *R. prowazekii* [19], and the neuronal network of the nematode *C. elegans* [20]. We find that the dependencies between certain features follow the same law for network models and for real data, thus justifying our approach.

Our third main result concerns one specific relation that was detected with our new method: we demonstrate that the synchronization index, a quantity introduced to assess the inertia to synchronization of complex networks [17], depends very strongly on the variance of the in-degree, a fact that may be of special interest for scientists studying network synchronization [10].

## Methods

### Models

Each of the network models (for a list of the models considered here see Table 1) is defined by a set of parameters; the rationale of the comparison is to first draw a random set of graph parameters, then draw a specific realization using these parameters, and finally analyze the structural properties of the graph. The parameters of most network models we analyze have to be chosen in a bounded set. It is therefore a natural choice to randomize the parameters using uniform (real or integer-valued) distributions. We will refer to this algorithm as to the doubly stochastic generation process. We kept the average connectivity (i.e. the expected fraction of realized edges out of all possible edges) fixed for all network models. In our study we used the value 0.1 throughout. This value generally resulted in relatively sparse networks with a large connected component. We concentrated our attention on directed networks, and, if necessary, we extended the original definitions to directed versions. For each realization of a network, we extract a feature vector 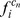 of commonly used statistical descriptors, see Table 2. The apex 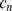 indicates the 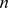 ^th^ instance of the network class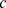, the index 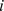 indicates the feature.

**Table 1:** Symbols and concepts.

Symbol	Description
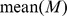	Complex number: mean of the set *M*
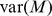	Positive real number: variance of the set *M*
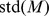	Positive real number: standard deviation of the set *M*
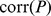	Real number in [−1,1]: Pearson correlation coefficient of pairs *P*
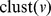	Real number in [0,1]: Fraction of undirected triangles between neighbors of *v*
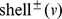	Positive integer: In or out-shell of node *v*
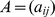	Matrix: adjacency matrix of a graph: *a_ij_* = 1 iff link 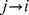 exists, otherwise 0
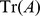	Complex, number: trace of the matrix *A*
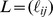	Matrix: Laplace matrix of a graph
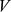	Set: node set of a graph
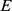	Set: edge set of a graph
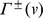	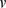 Sets of nodes: nodes targeting to or targeted by
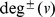	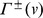 Integers: cardinality of
BA	Extended Barabási-Albert network
ER	Erdös-Rényi network
EQR	Equilibrium random networks
MF(n,k)	Multifractal network class: 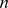 initial squares, 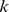 iterations
WS	Watts-Strogatz network

**Table 2:** Statistical descriptors (thematic ordering as in figures).

Symbol		Complete Name	Description
Local Descriptors			
CCM	Mean clustering		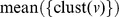
CCV	Clustering variance		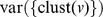
IDV	Variance of in-degrees		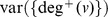
IOD	In-out correlation		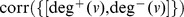
ODV	Variance of out-degrees		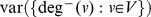
IPIC	In-mean-in correlation:		
	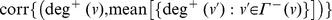		
IPOC	In-mean-out-correlation:		
	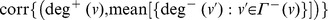		
OPIC	Out-mean-in-correlation:		
	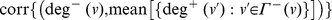		
OPOC	Out-mean-out-correlation:		
	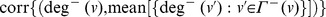		
FRC	Fraction of recurrent connections		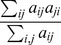
Global Descriptors			
SR	Spectral radius		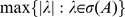
NTR	Normalized trace		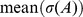
VEV	Variance of eigenvalues		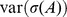
SI [17]	Synchronization index		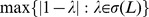
ST [10]	Synchronization time		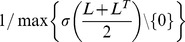
OSM	Mean of out-shells		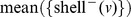
OSV	Variance of out-shells		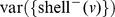
ISM	Mean of in-shells		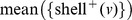
ISV	Variance of in-shells		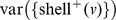
M	Modularity		See [21]

The descriptors were chosen such that many important aspects of complex networks are sufficiently covered, while keeping computational effort manageable. They can be subdivided in three categories:

- degree statistics: we consider average of in- and out-statistics, their fluctuations and several type of correlations;
- spectral statistics: we consider the spectral radius, average and fluctuations of the eigenvalue spectrum and two different synchronization measures;
- community structure: we consider average and fluctuations of the 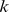-shell statistics and of the clustering coefficient, as well as Newman’s modularity.

We distinguish between “local” descriptors, which can be estimated by sampling small parts of the network, and “global” descriptors, for which knowledge of the full network is necessary. For example, to estimate the mean degree of the nodes in a network, it suffices to pick a number of nodes one after an other and count their neighbors. However, the spectral radius of the connectivity matrix is not the sum of spectral radii of small parts of the network, but depends on the structure of the whole network and therefore cannot be estimated in this way.

We use the same symbol (mean or var) for both the theoretical value and its unbiased estimation. Since the network parameters are independently chosen in every network realization, for fixed *c*, the numbers 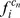 form a multivariate random variable whose realizations are independent over the instances *n*. As a consequence, dependencies between the 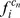 originate from statistical links across features.

### Feature Extraction

**Figure 1:**
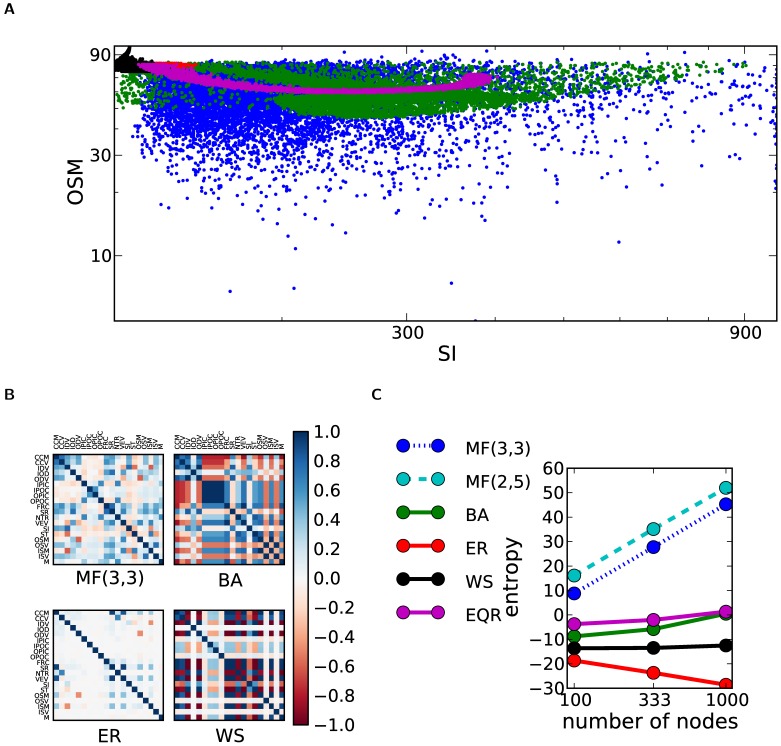
Variability generated by various network models. (a) Scattered data of two global features for realizations of different types of networks (size *N* = 1000), displayed in loglog scale. On the horizontal axis the synchronization index SI, on the vertical axis the mean out 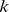-shell OSM of the corresponding graph are shown. (b) Correlations between pairs of features, arranged in a matrix (size *N* = 1000). For BA and WS networks, a clear structure is visible, due to the thematic ordering of the features. Strong correlations are, in fact, the major cause for the low feature entropy generated by non-MF networks, quantified in Panel (c). Entropy of the multivariate distribution of features. The feature entropy generated by MF networks is considerably higher, and it scales linearly with the number of nodes in the networks.

**Figure 2:**
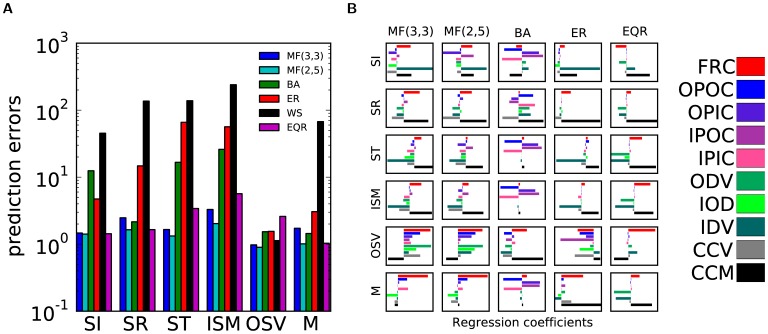
Prediction of global features from local ones. (a) Residual prediction errors. For the global features, we train a linear regression model with the data generated by one particular network model with random parameters and we test data from the remaining models. The residual prediction error is given by the mean-squared error normalized by the overall standard deviation of the corresponding feature. A value of 1 indicates the result obtained if the true mean of the population was known and used as a predictor. Note that using the empirical population mean as a predictor leads to a relative error larger than 1. MF network models perform consistently around 1, whereas other models have occasionally very large errors. (b) The coefficients of the linear regressor from the MF(3,3) set, normalized by the standard deviation of the local features used for the prediction. We excluded WS due to their very poor performance here. For some of the global features, the magnitude of the coefficients is consistent over the network models. For example, the positive contribution of the variance of the in-degree to the synchronization index and negative contribution to the synchronization time is consistent with the dynamic interpretation of these measures.

**Figure 3:**
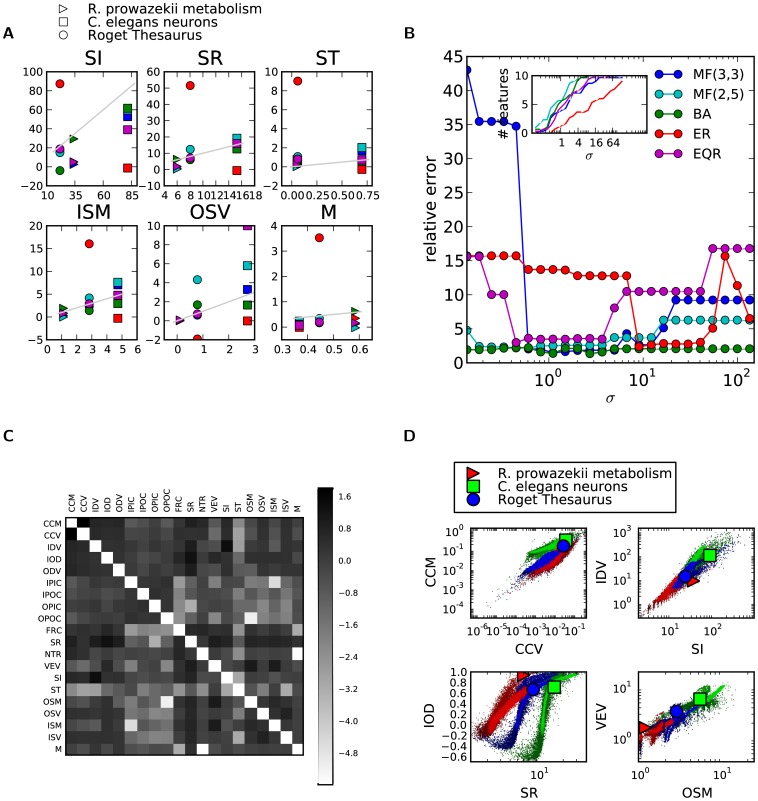
Prediction of global features in real-world networks. (a) Scattered data of the predicted global features for three data sets, using the regression coefficients obtained from network models with matched network size. Colors encode the model used for prediction. (b) To study whether the prediction is robust with respect to the chosen threshold, we depict the relative mean-squared error (defined as in Figure 2) averaged over the whole data-set of real-world networks as it depends on the threshold. The inset shows the average number of selected features for a given value of the threshold *σ*. (c) Reliability index 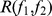 of the correlation coefficients between pairs of features, calculated across network models. High values point toward a general statistical law for all networks. (d) Data scatters for some pairs of features with significant correlations. Different colors encode different data sets: The number of nodes and the overall connectivity is extracted to generate a set of matched networks from various models. The scattered data are extracted from surrogate networks. The large markers denote the positions of the true data set in the data cloud. The statistics of the real-world networks lie in the data cloud, suggesting that those relations correspond to relevant statistical laws of complex networks. In the upper left panel, the *R. prowazekii* metabolism network is missing because of degenerate statistics.

For computing the statistics in Figures 1, 2 in we used 10 000 networks with 100, 333 or 1000 nodes, respectively, and with an overall connectivity of *p* = 0.1. For Figure 3 we used 4000 networks, where overall connectivity and node number were matched with the corresponding statistics of the real networks. We extracted the largest strongly connected component (LSCC) of each network using a classical algorithm [22]. All features were computed from the LSCC of the network. Typically, the LSCC equaled the whole network for classical network models or a large part of it in the case of MFs. Networks with a largest connected component of a size smaller than 0.1 times the number of nodes were discarded. Real data sets displayed different LSCC sizes: 274 (for 279 nodes, 2990 connections) for the *C. elegans* neural network, 413 (456 nodes, 1014 connections) for the *R. prowazekii* metabolic network and 904 (1022 nodes, 5075 connections) for the Roget synonym network. After the calculation of network features, networks with undefined features were discarded. A typical case occurred for Watts-Strogatz networks with low rewiring: if the degree sequence is constant, its variance is 0 and many correlation measures are undefined. Nevertheless, this occurred only rarely (less than 5 networks in 1000 generated ones).

### Erdös-Rényi Networks

These are the classical random networks [7]. Each connection is realized with probability *p*. Random networks of this type are, in fact, MF(1,1) networks. It must be noted that Erdös-Rényi networks do not have any free parameter in our study, since the connection probability is fixed. The only variability present in the Erdös-Rényi networks is due to the random realization of the edges and not to the parameter choice.

### Watts-Strogatz Networks

The Watt-Strogatz random network model [9] is constructed by connecting nodes on a ring up to a certain geodesic distance. Then a rewiring parameter 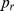 is chosen and every edge is randomly rewired with a probability *p_r_*. We started with a ring network with a given number of nodes. We then realized in-and out connections to 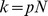 nearest neighbors such that the expected average degree is correct. Each connection is rewired to a randomly chosen target with a fixed rewiring probability *p_r_*, randomly chosen for every network as a uniform random real between 0 and 1.

### Extended Barabási-Albert Networks

Preferential attachment models like the Barabási-Albert models prescribe that, as nodes are added to the network, their connections are drawn randomly with a probability proportional to the degree of the target node.

For this study, we extend the classical preferential attachment model [8] in order to achieve a suitable randomization of statistics across networks. We also need to turn the graph into an oriented graph in such a way that the variances of the local features (across nodes) do not vanish. As a first step, we drew a uniform random integer of nodes between the mean degree 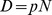 and the desired number of nodes *N*. Then, one node at the time was added, and bidirectional connections to existing nodes were established. Connection probability was proportional to the target degree, as in classical preferential attachment models. This procedure continued until the number of nodes reached *N*. Finally, we randomly break the network symmetry by deleting every edge independently with a certain probability which was chosen in order to obtain the final desired mean degree.

### Equilibrium Random Networks

Equilibrium random networks are characterized by a prescribed expected degree sequence. Nodes are then connected to each other with a probability proportional to the product of their expected degrees. This model has been introduced recently by Chung and Lu [15]. It differs slightly from the well known configuration model, where a network is constructed from a given degree sequence. A power-law degree sequence was generated with an exponent drawn uniformly between 0 and 4.

### Multifractal Networks

The multifractal network generator has been introduced recently by Palla et al. [13], [14]. The basic idea is that networks are created from a generating measure 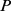 on the unit square with a complex and variable structure, leading to very variable networks. The generating measure is constructed in the following way: Initially, the interval (0,1) is divided randomly and uniformly into 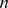 parts. Using divisions of the 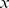 and 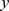-axes, the unit square is divided into 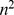 rectangles. The value of 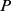 in each rectangle is drawn uniformly at random from the interval (0,1). In the next step, each rectangle is subdivided according to the initial division lengths, and the value of 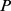 in each new rectangle is assigned from the initial probabilities, multiplied by the value of 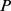 of the current rectangle. Thus, each rectangle is replaced by a shrunk version of the initial generating measure, times the value of 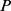 in the current rectangle. This procedure is repeated for 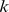 iterations, leading to an increasingly rough landscape, which for large 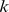 approximates a singular defining measure [23].

Once the generating measure 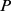 has been produced, to obtain a network with 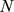 nodes and a desired mean degree *k*, we replace 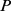 by
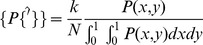
.

Each node 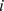 is then given a position 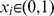 and a connection from node 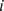 to 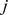 is made with a probability given by *P*(*x_i_*,*x_j_*). Deviating from the original proposal in [13], we do not impose a symmetry condition on 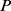 and draw each connection independently to obtain directed networks. Parameters are randomized by choosing random tuples of divisions lengths and probabilities.

## Results

### Variability of Networks Generated by Different Models

Feature variability and dependencies between features vary significantly between different network models. In fact, across our network samples, there are quite strong dependencies, as can be observed in Figure 1, Panel (a). For several of the network models, scattered feature pairs for realizations of networks with random parameters are concentrated in a small, specific area of the 2-dimensional feature space. Because feature pairs are not confined to these areas for alternative models, we conclude that these ensembles impose specific constraints. Therefore feature relations learned from these models cannot be generalized. We propose that an ensemble that is used to obtain relations that hold for a large number of networks should introduce as few dependencies as possible.

The dependencies can be quantified by computing the matrix of pairwise correlation coefficients between features, computed across realizations of the same network model. ER and MF networks have apparently the least correlated features, whereas EQR, BA and WS networks have features with strong correlations.

However, not only the correlations between features determine the intrinsic variability of a network model. The variability of the marginal distributions must also be considered. To discover relations between features that hold for a large set of networks, the network model should sample the space of networks completely and uniformly. However, the region of the feature space of a network model covered by a finite sample is necessarily bounded. The larger the variance of the features, the wider is the sampling of the model, and the larger is the set of networks where the inferred statistical relations are applicable.

We estimate the overall variability 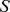 of a given class of networks generated by our doubly stochastic process by the logarithm of the determinant of the covariance matrix 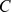 of the features,
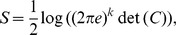
where 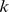 is the number of features. For an interpretation of this measure, assume that we approximate the distribution of features by a multivariate Gaussian distribution, where the covariances are given by the measured values. The Shannon entropy of this approximate distribution is given by *S*. In a geometrical interpretation, det (*C*) represents a measure for the volume of the feature space the network model is able to sample. It takes into account both the variability of the individual features as well as the loss in covered volume from correlated features. This measure is different from the entropy used in [24], [25], which depends on the discrete number of networks belonging to an ensemble. Our measure depends on the set of features, and the ability of a construction principle to sample the space of networks.

In Figure 1, Panel (c) it is apparent that the multifractal network generator (MF) by far outperforms all other network models with regard to feature variability. It is interesting to note that the variability of WS networks is considerably smaller than that of preferential attachment networks and equilibrium random networks. This is an important issue to keep in mind, especially in view of the large number of studies inspired by the Watts-Strogatz network model [26], [27]. The generated feature entropy reflects only partially the number of degrees of freedom of the network models. On the one hand, since the overall connectivity is fixed, ER networks do not have a single degree of freedom and they are the networks with the least generated feature entropy, whereas MF networks generate the largest feature entropy, also thanks to their larger number of degrees of freedom. However, on a finer scale, the generated feature entropy also depends on other factors. For example, the BA, EQR and WS network models all have one single degree of freedom, but the latter performs considerably worse. Furthermore, MF(3,3) have 10 degrees of freedom, but generate a lower feature entropy than MF(2,5), which only have 4 degrees of freedom.

### Predicting Global Features from Local Features

It has repeatedly been pointed out [5], [28] that local features of a network (e.g. degree distributions and degree correlations) are, when considered in isolation, not necessarily informative when it comes to predicting the dynamic properties of a network. On the other hand, global features (e.g. spectral properties and 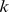-shell decomposition [5]) are difficult to obtain for large networks and, in general, are not robust against under-sampling of the network.

To overcome this problem, one could ask whether it is possible in principle to predict global features from a large set of simultaneously measured local ones. To test this idea, we trained for every network class a least-squares linear regressor on the vector of its local features to predict its global features. A distinct linear regressor was trained for every single global feature. As a test set we used the data set of networks of all classes with exception of the one used for the training of the linear regressor. In Figure 2, Panel (a) we compare the performance of the different network models. To this end, a prediction for the global feature 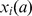 of a realization 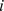 of a certain network type 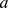 was calculated using the local features of the specific realization and the linear regression coefficients obtained from networks of type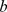. As a measure for the deviations between these values and the predictions 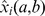 we consider the residual error
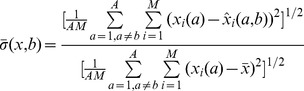
where 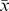 denotes the average of the feature across realizations and types, *M* indicates the total number of realizations of networks from each type and 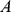 the number of network types. The normalization factor was included to make the performances for different features 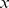 comparable.

Although least-squares linear regression is a rather simple approach to this complex problem, this procedure allows one to compare how well results from different network models can be generalized. Furthermore, interesting information can be extracted from an examination of the regression coefficients, see Figure 2, Panel (b) and our discussion below.

Finally, we studied whether our approach can be applied to real-world networks extracted from publicly available data sets. We considered the connectome of the nematode *C. elegans* [20], a synonym network based on the Roget’s Thesaurus retrieved from the Pajek data sets collection [18], and the metabolic network of the bacterium *R. prowazekii* [19]. Our selection was based on several criteria: first, their size matched the size of the networks used for the evaluation of variability. Furthermore, they represent directed graphs and have a large strongly connected component. Finally, their physical/biological nature is quite diverse. For each of the data sets we generated a sample set of networks as described above, with matched number of nodes and average connectivity. On each network data set, we trained a linear regression model using an appropriate subset of local features. The subset was chosen such that local features not represented well in the data set are excluded. To this end, we fixed a threshold 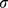 and only used those local features whose value did not deviate from the average value of the corresponding training set by more than 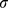 standard deviations. For each data set we studied how the regression performance depends on the threshold. The performance was quantified by the relative mean-squared error calculated across global features and networks, see Figure 3. For this purpose, all of the MF, EQR and BA networks resulted in regression models with quite good predictive power. This is demonstrated in Figure 3 (a), where predicted features are similar to the measured ones, indicated by positions of the scattered symbols close to the diagonal. Figure 3 (b) shows the dependency of the prediction error on the subset of features used for prediction. As expected, a larger number of local features increases the predictive power, as long as the corresponding feature of the real data is well represented in the model data set.

Furthermore, it is possible to use real networks as a cross-validation for the statistical methods we are proposing. To this aim, we first want to estimate the reliability of the correlation between two features. This is done by computing a 2-dimensional matrix with the entries
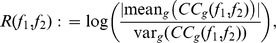
where 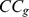 is the matrix of correlation coefficients between features in network class *g*. This matrix, depicted in Figure 3, Panel (c), assesses the reliability of a correlation between two features across models. It takes into account both the absolute size of the correlation as well as its consistency across different models. The ten relations with the highest reliability index are listed in Table 3.

**Table 3:** Correlated feature pairs with highest reliability index.

Feature 1	Feature 2
CCV	CCM
SI	IDV
SR	IOD
OSM	VEV
ISM	VEV
VEV	SR
SR	IDV
SR	ODV
SR	CCM

To decide whether the relations between features are a peculiarity of the stochastic network models under consideration, we compare the model statistics with the true data previously introduced. In scatter plots of the feature pairs with the highest 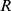 values, statistical relations between the two features impose constraints on the area that is accessible for data points, if network parameters like size and connectivity are fixed, Figure 3, Panel (d). If a relation between two features is of the same type both in real-world and model networks, then one would expect that the feature pair for the real-world network lies on the corresponding manifold for the model networks. Indeed, we verify in a scatter plot that the true data lie on the same manifold as the model data. We can thus conclude that a high 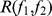 value is a good predictor of the reliability of the correlation between a feature pair *f*_1_,*f*_2_. This cross-validation method allowed us to reveal statistical laws for networks that would otherwise be quite difficult to discover. Three selected examples are highlighted below and, in the following paragraph, we discuss the synchronization properties of networks in greater detail.

1. Mean and the variance of the clustering coefficient over the network are consistently (positively) correlated across networks (mean Pearson’s correlation 0.79, standard deviation 0.12). As a consequence, properties attributed to the mean clustering coefficient [29], [30] could be as well attributed to the variance of the clustering coefficient. In this type of studies, additional considerations must be taken into account to disentangle the contributions of these two measures.
2. The variance of the distribution of the eigenvalues (seen as a complex-valued random variable) is consistently (positively) correlated with both the mean of the in- (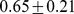) and the out-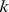-shell decomposition (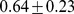). The mean in- and out 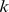-shells encode, roughly speaking, how well-connected the network is. Local 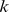-shell values are, as an example, predictive for epidemic spreading efficiency [5]. We thus speculate about a role for eigenvalue variance in determining the connectedness of a complex network. Although this observation is purely heuristic, it could be of help for scientists who use 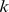-shell decompositions as a tool to understand the dynamics of complex networks.
3. The spectral radius is consistently (positively) correlated with the mean clustering coefficient (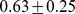), with the variance of the in-degree and the variance of the out-degree (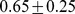 in both cases), and with the in-out degree correlation (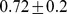). The latter has an intuitive interpretation: the spectral radius is related to the stability properties of an associated linear system. The spectral radius 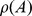 determines the asymptotic behavior of the linear dynamical system defined by the recurrence equation *x_n_*_+1_ = *Ax_n_*. A high in-out degree correlation means that nodes receiving input from many inputs project to many other nodes, thus destabilizing the system. Finally, the spectral radius is, as expected, consistently (positively) correlated with the eigenvalue variance (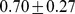).

### Synchronizability and In-degree Variance

The two features “synchronization index” and “in-degree variance” are a very interesting case that deserves special attention. The synchronization index has been introduced for directed graphs to quantify the degree to which a network is prone to synchronization [17]. Low values of this index indicate that the networks synchronize easily.

For MF, EQR and BA networks multivariate linear regression is most efficient, and for these models the synchronization index and the in-degree variance have a correlation coefficient of 0.85±0.04. This is in marked contrast to the fact that these networks are of very different character: MF and EQR are locally of Erdös-Rényi type, whereas BA is not; MF and BA networks typically have narrow unimodal SI distributions, whereas EQR networks exhibit a peculiar uniform SI distribution. EQR and BA have a degree distribution with power-law tails, a property not shared by MF networks.

Our observations are in contrast to the conclusions previously drawn [17] regarding the difficulty of predicting synchronizability by statistical network properties. Our results imply that, for real-world networks, statistical properties can indeed be informative about spectral properties. We also have shown that local statistical properties, as the variance of the in-degree, can be used to infer spectral properties. It must be mentioned that related results have been analytically obtained for the case of undirected networks [16]. These results extend the observation by Grabow et al. [10] that networks in the small-world regime with fixed in-degrees synchronize slowly.

In the framework of small world networks it has further been suggested [9] that networks with a high clustering coefficient (CCM) have a small synchronization index (SI). As our analysis shows, this relation is not conserved across network models: In fact, all network models apart from WS that we consider here show the inverse of the proposed relation, namely a positive correlation of CCM and SI. Apparently only in WS networks clustering is beneficial for synchronization.

Furthermore, our results are consistent with recent results obtained in the theory of neuronal networks [31]. There, it has been shown that in a network model similar to our EQR setting, decreasing the variance of the in-degree distribution leads to fast oscillations.

## Discussion

A significant amount of recent research has focused on non-random aspects of real biological networks, especially in studies of metabolic interactions [19], of neuronal networks [20], [32], [33], and of epidemic spreading [5]. In neuroscience, in particular, the question has arisen of how various network features influence network performance with respect to different computational aspects [34], [35]. In this type of works, different approaches have been used. The first approach is to use data from related real-world data sets [5], [36], [37]. One difficulty presented by this approach the generate surrogate data. Degree preserving randomization has been suggested as a method for assessing statistical significance of observed features in this approach [38]–[40].

Alternatively, *ad hoc* network models have been developed for studying the effect of specific network features on the model dynamics [11], [30], [41], [42]. In this work, we assessed the generalization power offered by commonly used network models. According to our analysis, a crucial limitation of most of the currently used network models is their low statistical variability in the network features exhibited by the generated networks. This makes it unlikely that results obtained for a specific network model can be extrapolated to other contexts.

In particular, the often employed WS (“small-world”) model has quite singular statistical properties; on the one hand, the feature entropy generated by WS networks with randomized wiring parameter is, at least for small networks, only slightly larger than the entropy generated by ER graphs, which have no free parameter when the mean connectivity is fixed. In fact, ER networks are a special case of WS networks where the rewiring parameter is 1. On the other hand, WS graphs are outperformed by EQR networks with a randomized exponent of the degree distribution, which also have one degree of freedom, increasing the feature entropy of the ensemble. It finally should be mentioned that the EQR model has some points in common with the degree preserving randomization algorithm proposed by Milo and coauthors [38].

We found that the MF network generator [13], [14] offers the possibility to generate quite variable random networks with high predictive power. The feature entropy of the graph ensemble defined by these models is higher than the one generated by BA, EQR, WS and ER models. This property is due to the efficient use of a larger number of degrees of freedom in the network generating algorithm. Moreover, in contrast to other types of networks, the feature entropy of the ensemble seems to scale linearly with the size of the networks in this case. This property allows one to reliably learn relations between local and global network features. We demonstrated that these relations indeed encompass predictive power also for real world networks up to the point that global properties can be predicted from local ones. This, however, is only possible if the local features of the real world networks are well represented in the ensemble defined by the network model. This fact once again highlights the importance of networks with broad distributions of many features.

Finally, and most importantly, we collected specific pieces of information regarding network properties by numerical experimentation. A striking example concerns the negative correlation of the variance of in-degrees with network synchronizability. Results in this direction have already been obtained [31], [43], although on specific topologies obtained with an algorithm similar to EQR. Our results indicate that this may be a rather general property of dynamical systems on networks. This finding could have important consequences, especially in view of the increasing evidence for a link between structural heterogeneity and stability in complex networks. Our method can be applied to include additional network features, like motif distributions, or characteristics of dynamical systems on networks, and we would expect that further dependencies can be discovered between that have escaped our attention so far.
